# G2019S Variation in LRRK2: An Ideal Model for the Study of Parkinson’s Disease?

**DOI:** 10.3389/fnhum.2019.00306

**Published:** 2019-09-04

**Authors:** Chao Ren, Yu Ding, Shizhuang Wei, Lina Guan, Caiyi Zhang, Yongqiang Ji, Fen Wang, Shaohua Yin, Peiyuan Yin

**Affiliations:** ^1^Department of Neurology, The Affiliated Yantai Yuhuangding Hospital of Qingdao University, Yantai, China; ^2^Department of Neurology, The Second Affiliated Hospital of Soochow University, Suzhou, China; ^3^Institute of Neuroscience, Soochow University, Suzhou, China; ^4^Department of Orthopedic Surgery, The First Affiliated Hospital of Soochow University, Suzhou, China; ^5^Department of Neurosurgical Intensive Care Unit, The Affiliated Yantai Yuhuangding Hospital of Qingdao University, Yantai, China; ^6^Department of Emergency and Rescue Medicine, Xuzhou Medical University, Xuzhou, China; ^7^Department of Nephrology, The Affiliated Yantai Yuhuangding Hospital of Qingdao University, Yantai, China; ^8^Department of Nursing, The Affiliated Yantai Yuhuangding Hospital of Qingdao University, Yantai, China; ^9^Department of Blood Supply, Yantai Center Blood Station, Yantai, China

**Keywords:** LRRK2, G2019S mutation, Parkinson’s disease, disease model, pathogenesis

## Abstract

Parkinson’s disease (PD) is the second most common neurodegenerative disorder and has plagued humans for more than 200 years. The etiology and detailed pathogenesis of PD is unclear, but is currently believed to be the result of the interaction between genetic and environmental factors. Studies have found that PD patients with the LRRK2:G2019S variation have the typical clinical manifestations of PD, which may be familial or sporadic, and have age-dependent pathogenic characteristics. Therefore, the LRRK2:G2019S variation may be an ideal model to study the interaction of multiple factors such as genetic, environmental and natural aging factors in PD in the future. This article reviewed the progress of LRRK2:G2019S studies in PD research in order to provide new research ideas and directions for the pathogenesis and treatment of PD.

## Background

Parkinson’s disease (PD) is the second most common neurodegenerative disorder after Alzheimer’s disease, and has plagued humans for more than 200 years. According to statistics, the prevalence of PD is approximately 0.3% in developed countries and is 1% in individuals over 60 years old. The main pathological changes in PD are the formation of Lewy corpuscles and a decrease in dopaminergic neurons (DANs) in the substantia nigra-striatum system, which leads to a decrease in dopamine (DA) content in the related nerve endings and an imbalance between DA and acetylcholine. It is generally believed that when DANs in the substantia nigra are reduced by more than 50% and DA content is reduced by more than 70%, PD patients will exhibit typical motor symptoms such as movement retardation, static tremor, myotonia and abnormal posture and gait (Rogers et al., 2017). Of course, prior to that, other non-motor symptoms may also occur in some PD patients such as sensory disturbance and sleep disorders^1^. Clinically, PD can be divided into sporadic PD and familial PD. The etiology of sporadic PD is unclear. Familial PD may be caused by gene mutation. The age at onset, the rate of progression and the severity of PD vary, which may be the result of the interaction between genetic and environmental factors (Pan-Montojo and Reichmann, 2014). However, the true real etiology and full pathogenesis of PD are still unclear. At present, the incidence of PD increases with the aging of the population and an increased life span, and the prevalence of PD is also rising worldwide. Because of the large population in China, the increasing number of PD patients has resulted in heavy economic and psychological burdens to society and families. Although scientists continue to make efforts to study the diagnosis and treatment of PD, few novel and significant breakthroughs have been reported. Therefore, there is an urgent need for more in-depth innovative research on the pathogenesis of PD to obtain more effective and updated intervention and prevention methods.

With the identification and cloning of disease-related genes, such as the *leucine-rich repeat kinase 2* (LRRK2), α*-synuclein* (αSyn), SNCA, Parkin, PINK1, and GBA, the role of genetic factors in PD has attracted more attention (Lee and Liu, 2008). At present, of the 23 known pathogenic genes of PD, only LRRK2 is associated with both sporadic and familial PD. In addition, PD patients with the LRRK2 variation often present all the major clinical manifestations of typical non-carrier PD patients. Therefore, it is of great significance to carry out relevant research on PD patients with the LRRK2 variation (Di Maio et al., 2018). Among LRRK2 variations, G2019S mutation is the most common, as seen in familial and sporadic PD. In addition, the penetrance of LRRK2:G2019S mutation in PD is age-dependent, which suggests the important involvement of age and environmental factors (Goldwurm et al., 2007). This is consistent with the hypothesis that PD is attributed to the interaction of genetic, environmental, natural aging and other factors. Therefore, the LRRK2:G2019S mutation may be an ideal disease model and one potential breakthrough in PD research to determine the mechanisms of PD and to develop new treatment methods by studying PD patients with LRRK2:G2019S variation.

## Introduction of LRRK2

**FIGURE 1:**
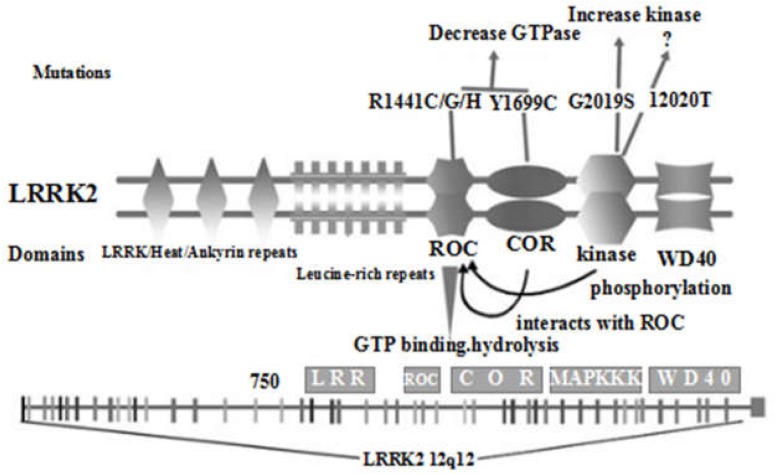
Schematic diagram of the distribution of the main domains of LRRK2 protein and the location of current important mutations (Re-creation based on Cookson, 2010 and Mata et al., 2005).

The coding gene of LRRK2 is located on chromosome 12q12, spans 7,584 bp, and contains 51 exons. The LRRK2 protein weighs 280 kD, has 2,527 amino acids, and consists of ankyrin-like repeats (ALRs), leucine-rich repeats (LRRs), the Ras of complex (Roc) GTPase domain, carboxy-terminal of Roc (COR), kinase domain, and the WD40 domain from the N-terminal to C-terminal (Mata et al., 2006). LRRK2 is expressed in brain tissues such as the brain stem (midbrain), striatum, olfactory bulb, cortex, hippocampus, and cerebellum. LRRK2 is mainly involved in the regulation of protein translation, axon growth and aging in the nervous system, and its mutation was discovered in 2004 (Zimprich et al., 2004). At present, the functions of the major domains of LRRK2 have not been fully defined. It is generally believed that the WD40 domain, LRR sequence and ALR sequence are involved in protein-protein interactions (Figure 1; Gloeckner et al., 2006; Ito et al., 2007). In addition, the WD40 domain may be involved in protein-lipid interactions, suggesting a possible interplay between LRRK2 and membrane structures. The tandem COR domain and Roc domain are unique to and prevalent in the ROCO superfamily of proteins. The interaction between COR and Roc domains facilitates the formation of dimers among ROCO proteins. Normally, LRRK2 proteins act as dimers, the formation of which is dependent on the COR domain. Sequence homology analysis and functional characterization demonstrated high sequence similarity between LRRK2 and mixed-lineage kinases (MLK). However, MLK possess serine/threonine and tyrosine kinase activity, while LRRK2 has no tyrosine kinase activity. MLK belongs to the mitogen-activated protein kinase (MAPK) family, exerting the function of a MAPK kinase kinase (MAPKKK). Despite the high similarity between LRRK2 and MLK, whether LRRK2 is also a MAPKKK and how its role as a MAPKKK is played are still unclear, as the activation pathway of LRRK2 and its downstream kinase effectors are currently unknown. Proteomics and random peptide analysis have suggested that LRRK2 is a serine/threonine protein kinase that preferentially phosphorylates threonine.

Relevant studies (Di Fonzo et al., 2005; Gilks et al., 2005; Mata et al., 2005; Nichols et al., 2005) have found that there are many mutation sites in the *LRRK2* gene, resulting in approximately 100 LRRK2 variants, many of which are related to PD, and the G2019S, R1441G, R1441C, R1441H, Y1699C, I2020T, and N1437H variants were confirmed to be pathogenic. Among these variants, for R1441G, R1441C, and R1441H the mutation is located within the Roc GTPase domain, while for Y1699C the mutation is within the COR domain, which also reduces the Roc GTPase activity. In addition, the newly discovered N1437H mutation within the Roc GTPase domain is also pathogenic. However, it is not clear which specific effects it has on GTPase activity. Finally, the remaining I2020T and G2019S mutations are located in the kinase domain, which increases the kinase activity of LRRK2. Recently, new mutations such as G2385R, A1441G and R1628P have also attracted considerable attention.

## LRRK2:G2019S and PD

Among the variations of LRRK2, G2019S is the most common. It not only comprises a high proportion (approximately 4–5%) in familial PD, but is also a common mutation (approximately 1%) in sporadic PD. Furthermore, it is markedly increased in specific populations and is up to 40% in North Africans and 30% in Ashkenazi Jews (Trinh et al., 2016). It is the first example of a Mendelian form of PD^2^. This variation originated from a G > A substitution at position 6055 of exon 41 of LRRK2 gene that results in the change of a glycine to serine at codon 2019 of LRRK2. It was previously believed that the LRRK2:G2019S variation mainly occurred in Caucasians and no correlation was found between G2019S and PD in Asians until one such case was reported in Japan (Pirkevi et al., 2009). Fundamental studies (Xiao et al., 2015, Karuppagounder et al., 2016; Howlett et al., 2017; Kim et al., 2017, 2018; Litteljohn et al., 2018; Vermilyea and Emborg, 2018) revealed that the LRRK2:G2019S variation can lead to elevated levels of αSyn and tau proteins, mitochondrial dysfunction, synaptic vesicle transport disorder, and can induce abnormal Erk, c-Jun and Akt signaling pathways, leading to apoptotic regulation disorder and hyperautophagy, reduce neurite growth, increase abnormal growth and differentiation of DANs cells, and induce cellular degeneration. However, the specific structural and functional changes in DANs and their related influencing factors and mechanisms in LRRK2:G2019S-bearing PD patients are still unclear.

Similar to many other LRRK2-associated PD patients, PD patients with G2019S mutations have a heterogeneous pathology. However, the pathology has been reported in patients with a G2019S mutation, and it not only conforms to the typical α-synuclein Lewy-body type of PD, but also include diffuse Lewy-body disease, nigral degeneration without distinctive histopathology and, rarely, even aggregates of the microtubule-associated protein tau, suggestive of progressive supranuclear palsy or frontotemporal dementia. To date, it is still not fully understood how LRRK2 may affect the biology/pathobiology of α-Syn. One possibility is that the effect of LRRK2 on α-Syn may take place via the modulation of other key proteins, such as Rab GTPases or other kinases, which affect pathways involved in the degradation of α-Syn, and the propagation of pathology as well, resulting in PD-associated features, for example the appearance of typical α-synuclein Lewy-bodies (Outeiro et al., 2019). Accordingly, a question is raised: “Is LRRK2 detection in human biofluids a potential Parkinson’s disease biomarker?” (Taymans et al., 2017). The answer is no. Although LRRK2 cannot be used as a biomarker for PD, West (2017) suggested that recommendations should be given for a biomarker-guided initial entry of LRRK2 kinase inhibitors in PD patients. Of course, this also includes those patients with a G2019S mutation. Interestingly, the total neopterin levels in the cerebrospinal fluid (CSF) of the LRRK2-PD patients may be one of the candidate biomarkers, which might be useful for understanding the pathophysiology of patients with a G2019S mutation (Ichinose et al., 2018).

Studies have found (Belarbi et al., 2010; Alcalay et al., 2013; Gatto et al., 2013; Sierra et al., 2017; Gunzler et al., 2018; Mestre et al., 2018) that the clinical features of PD patients with the LRRK2:G2019S variation included a high average age at onset, more female patients, long disease course, starting mainly in the lower limbs, abnormal posture and gait disorders, and more depression, hallucinations, sleep disorders and cognitive disorders. The remaining core clinical features of PD patients carrying the variation are similar to those of PD patients not carrying the variation. Moreover, the penetrance in LRRK2:G2019S carriers increases from only 28% at 59 years of age to 51% at 69 years, suggesting that it is age-dependent. It has been reported that dynamin 3 (DNM3) may be a potential genetic modifier in the relationship between LRRK2 and age-dependent penetrance in LRRK2-associated PD in Arab-Berber patients (Trinh et al., 2016). However, recent studies in Spain (Fernández-Santiago et al., 2018), Asia (Foo et al., 2019) and China (Yang et al., 2019) have not yielded similar results. An incidental finding showed that SNCA but not DNM3, modifies the age at onset of LRRK2-related PD, and the studies in Spain (Fernández-Santiago et al., 2018) and China (Yang et al., 2019) were noteworthy.

The information mentioned above is in accordance with the current hypothesis that PD is caused by the interaction of genetic factors, environmental factors and natural aging factors. Therefore, this will promote the future study of PD if a PD model with the LRRK2:G2019S variation can be established.

The development of induced pluripotent stem cells (iPSCs) and related techniques has provided new ideas for resolving the above issues (Park et al., 2008; Bumpei et al., 2016). iPSCs are generated by introducing pluripotency-related factors such as Oct4, Sox2, Klf, and c-Myc into mature somatic cells, so that they can be reprogrammed and restored to the cell state with embryonic stem cell characteristics. These cells can differentiate into cell types of multiple lineages (Chari and Mao, 2016).

With maturation of the iPSCs technology, the establishment of an iPSCs cell model from a LRRK2:G2019S PD patient was reported in 2011 (Nguyen et al., 2011), and related publications have gradually increased (Liu et al., 2012; Mak et al., 2012; Reinhardt et al., 2013; Schwab and Ebert, 2015). However, up to now, there has been no report on the iPSCs cell model derived from the LRRK2:G2019S mutant lineage with the same genetic background. Therefore, it will be of great significance to obtain the LRRK2:G2019S mutant lineage with the same genetic background and to use iPSCs and related technologies to study the pathogenesis of PD in patients with LRRK2:G2019S mutation. This can then be used to develop relevant prevention and treatment strategies, especially when there are PD patients and non-onset carriers present in the family.

## Conclusion: Questions and Prospects

The onset of PD caused by the LRRK2:G2019S variation is the outcome of interactions between multiple genes and molecular mechanisms. On the one hand, this involves the intersection of familial and sporadic PD, and on the other hand the embodiment of PD gene-environment-aging factor interactions. Therefore, more attention should be paid to the study of LRRK2:G2019S variation. It has been suggested that individuals with a family history of PD caused by LRRK2:G2019S variation should be screened. Early dopamine transporter imaging with single photon emission computed tomography (DAT-SPECT) evaluation (Artzi et al., 2017) or measurement of Lamp2 concentration in the cerebrospinal fluid has been proposed (Klaver et al., 2018). However, further investigations are necessary to determine whether these procedures are applicable to areas with a low incidence and a low LRRK2:G2019S mutation rate, as a LRRK2:G2019S mutation carrier who was neurologically healthy at the age of 80 has been reported (Kay et al., 2005). Of course, the study of LRRK2:G2019S-related PD is not only about disease screening and diagnosis, but also about its clinical application in the future. It was found that the effect of deep brain stimulation (DBS) in patients with LRRK2 gene was better than that in non-mutation carriers (Sayad et al., 2016).

In addition, research on the genetic correction of LRRK2: G2019S has also been carried out (Sanders et al., 2014). At present, most of the targeted drugs are LRRK2 kinase inhibitors (Deng et al., 2011). Other drugs include coenzyme Q10, rapamycin and lovastatin (Cooper et al., 2012; Lin et al., 2016). Finally, we believe that the LRRK2:G2019S mutation will not only open a novel era in PD genetics, as proposed by Bonifati (2006), but will also bring about a new prospect, as the latest research showed that normal *LRRK2* gene also promotes PD (Di Maio et al., 2018).

## Author Contributions

CR, YD, and SW found references and drafted the manuscript. YD and SW readed literature. LG and CZ summarized information. YJ helped to draft the manuscript. FW, SY, and PY designed literature retrieval strategy. CR, FW, SW, and SY modified and revised the manuscript. YD and PY drew figure. CR obtained fundings. All authors read and approved the final manuscript.

## Conflict of Interest Statement

The authors declare that the research was conducted in the absence of any commercial or financial relationships that could be construed as a potential conflict of interest.

## Footnotes

1 www.nice.org.uk/guidance/ng71

2 http://www.intechopen.com/books/etiology-and-pathophysiology-of-parkinson-s-disease/genetics-of-parkinsondisease
